# Cold Adapted *Nitrosospira* sp.: A Potential Crucial Contributor of Ammonia Oxidation in Cryosols of Permafrost-Affected Landscapes in Northeast Siberia

**DOI:** 10.3390/microorganisms7120699

**Published:** 2019-12-14

**Authors:** Tina Sanders, Claudia Fiencke, Jennifer Hüpeden, Eva Maria Pfeiffer, Eva Spieck

**Affiliations:** 1 Helmholtz Zentrum Geesthacht, Institut für Küstenforschung, Max-Planck-Str. 1, 21502 Geesthacht, Germany; 2 Universität Hamburg, Institut für Bodenkunde, Allende-Platz 2, 20146 Hamburg, Germany; Claudia.Fiencke@uni-hamburg.de (C.F.); Eva-Maria.Pfeiffer@uni-hamburg.de (E.M.P.); 3 Center for Earth System Research and Sustainability, Universität Hamburg, Allende-Platz 2, 20146 Hamburg, Germany; 4 Universität Hamburg, Mikrobiologie und Biotechnologie, Ohnhorststr. 18, 22609 Hamburg, Germany; Jennifer_huepeden@gmx.de (J.H.); eva.spieck@uni-hamburg.de (E.S.)

**Keywords:** permafrost-affected landscapes, cryosols, canonical nitrification, *Nitrosospira*, ammonia oxidizers, cold environment, Arctic

## Abstract

Permafrost-affected landscape soils are rich in organic matter and contain a high fraction of organic nitrogen, but much of this organic matter remains inaccessible due to nitrogen limitation. Microbial nitrification is a key process in the nitrogen cycle, controlling the availability of dissolved inorganic nitrogen (DIN) such as ammonium and nitrate. In this study, we investigate the microbial diversity of canonical nitrifiers and their potential nitrifying activity in the active layer of different Arctic cryosols in the Lena River Delta in North-East Siberia. These cryosols are located on Samoylov Island, which has two geomorphological landscapes with mineral soils in the modern floodplain and organic-rich soils in the low-centered polygonal tundra of the Holocene river terrace. Microcosm incubations show that the highest potential ammonia oxidation rates are found in low organic soils, and the rates depend on organic matter content and quality, vegetation cover, and water content. As shown by 16S rRNA amplicon sequencing, nitrifiers represented 0.6% to 6.2% of the total microbial community. More than 50% of the nitrifiers belonged to the genus *Nitrosospira*. Based on PCR *amoA* analysis, ammonia-oxidizing bacteria (AOB) were found in nearly all soil types, whereas ammonia-oxidizing archaea (AOA) were only detected in low-organic soils. In cultivation-based approaches, mainly *Nitrosospira-*like AOB were enriched and characterized as psychrotolerant, with temperature optima slightly above 20 °C. This study suggests a ubiquitous distribution of ammonia-oxidizing microorganisms (bacteria and archaea) in permafrost-affected landscapes of Siberia with cold-adapted AOB, especially of the genus *Nitrosospira,* as potentially crucial ammonia oxidizers in the cryosols.

## 1. Introduction

Tundra landscapes and their cold soils (cryosols) play an important role in the global carbon (C) and nitrogen (N) cycle. Besides representing a large pool of C, they also retain more than twice as much N as temperate soils [1,2]. While tundra soils—mostly permafrost-affected soils—store high amounts of nitrogen in organic fractions, the ecosystem remains nitrogen-limited because only a small fraction of total nitrogen is available as inorganic N (DIN) forms, such as ammonium (NH_4_^+^) and nitrate (NO_3_^–^) or dissolved organic nitrogen (DON) [3,4]. In contrast to temperate systems, Arctic N-limitation is caused by very low N-input by N-atmospheric deposition [5,6] and very minimal N-fixation by free-living N_2_-fixing bacteria or cyanobacteria in lichen or bryophytes [6,7,8]. Furthermore, microbial decomposition of organic matter, and therefore N-mineralization, is restricted by low temperatures, high water saturation conditions, a short vegetation period, lower litter quality, high CN ratio, and hence low nutrient availabilities [4,9,10,11,12]. Since topography in arctic terrestrial ecosystems influences these environmental control factors, the N-cycle and N-availability in permafrost-affected soils is driven by arctic microscale and topographic variations [6,13,14]. In these N-limited ecosystems, the N-availability is an important key driver of the N- as well as C-cycle and the balance between primary production and soil organic matter composition. Additionally, it influences the formation of the climate relevant gases such as carbon dioxide (CO_2_), methane (CH_4_), and nitrous oxide (N_2_O), which will increase because of the warming of subarctic tundra [12,15,16,17]. 

Due to low N-availability in these vegetated soils, reactive N is rapidly internally recycled, causing a minimal release of nitrate or nitrous oxide [15,18]. Availability of reactive N, such as DIN and DON, is affected by aerobic nitrification, anaerobic denitrification, and anaerobic ammonium oxidation (anammox), and all these processes are microbially catalyzed [19]. Although nitrification—the microbiological oxidation of ammonia to nitrite and further to nitrate—has fundamental importance for the N-cycle and controls the N-availability in these terrestrial ecosystems, nitrification has not been comprehensively investigated in permafrost-affected soils [6,20,21,22,23,24,25]. The first step of nitrification—ammonia oxidation—is performed by ammonia-oxidizing bacteria (AOB) and archaea (AOA), while the second step is carried out by nitrite-oxidizing bacteria (NOB). However, recent studies have shown that both steps of nitrification can be performed by a single microorganism, the NOB *Nitrospira* called comammox [26,27].

Terrestrial AOBs are classified into the genera *Nitrosomonas* and *Nitrosospira* within the Betaproteobacteria family. *Nitrosospira* is ubiquitously distributed [28,29] and is the primary genus of AOBs in soils [30]. It is most often found in natural habitats such as forests, acidic soils, and cryosols [31,32,33,34]. A cold-adapted *Nitrosospira lacus* sp. nov. from sandy lake sediment has been previously described [35]. In cryosols, AOBs are often present in high abundance but low diversity, with the spatial distribution depending on differences in soil parameters [23,36,37]. In comparison to AOB, AOA of phylum Thaumarchaeota [38] are phylogenetically more widespread and are affiliated with different genera, for instance, *Nitrosopumilus*, *Nitrososphaera*, *Nitrosocaldus*, and *Nitrosotalea* [37,39]. The ecological significance of AOB versus AOA is controversial and not fully understood [40,41,42], especially in arctic environments [23,24,25,42,43,44,45]. Recently, this discussion has been extended to the role of comammox in the nitrogen cycle [46,47]. Ammonia oxidation is positively influenced by oxygen and ammonium availability, which is coupled with the quality of the organic matter content, as well as pH and water content [42]. In particular, pH seems to be a key ecological factor controlling the composition of soil ammonia oxidizer communities [48]. Furthermore, Arctic ecosystems are especially sensitive to changes in temperature [49]. Extreme temperatures and short vegetative periods play important roles in Arctic nitrogen cycling.

The objectives of this study are to investigate (1) the presence of different groups of nitrifiers in the microbial community, (2) the potential net nitrification rates in the different soil types of Samoylov Island, which differ mainly in organic matter content, (3) the temperature dependence of nitrification rates in two different soils of the polygonal tundra and the beach, and (4) the temperature adaptation of nitrifying enrichment cultures, containing *Nitrosospira*. We applied two main approaches to address these questions. First, soil samples acquired during an expedition in summer 2008 were analyzed for microbial community composition, and microcosm incubations were done to determine potential nitrification rates. Second, nitrifiers were enriched from soil samples of these sites to characterize them and their temperature adaptation.

## 2. Materials and Methods 

### 2.1. Investigation Site

**Figure 1:**
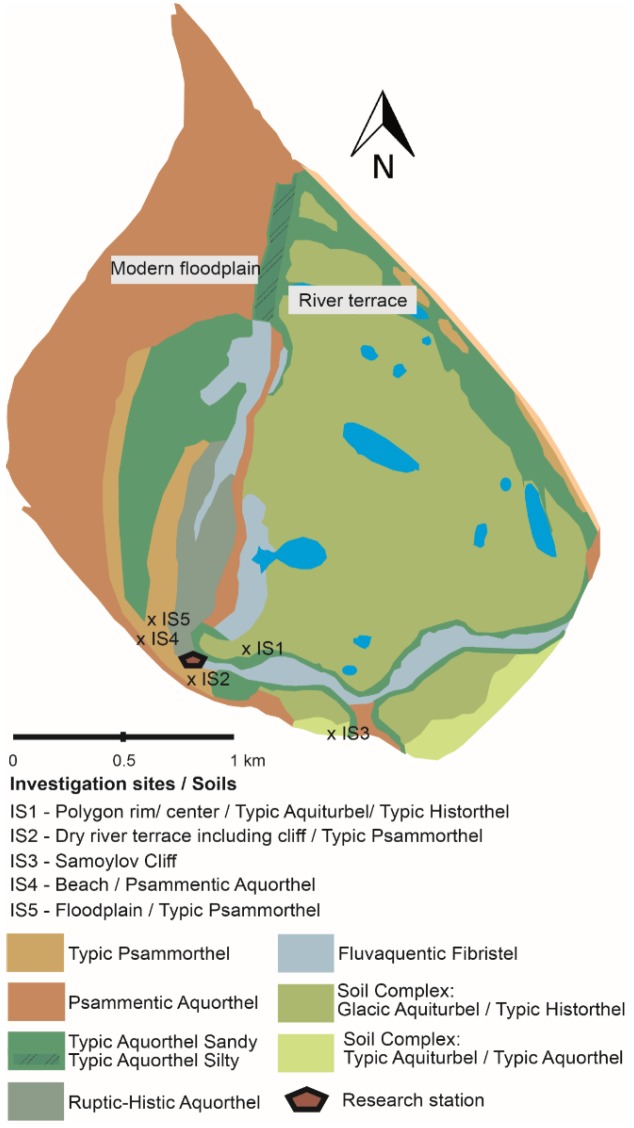
Soil map of Samoylov Island with indicated geomorphological units: in the western part, the “modern floodplain”, and in the eastern part, the “river terrace” with polygonal tundra [53].

The Lena River Delta is located in northeastern Siberia, where the Lena River discharges into the Laptev Sea as part of the Arctic Ocean. The study site is located in the youngest part of the Lena Delta on Samoylov Island (N 72°22, E 126°28) [50]. In the zone of continuous permafrost, the mean annual air temperature (MAAT) is –13.5 °C, the mean annual precipitation (MAP) is 200 mm [51], and the mean soil temperature is –8.4 °C. In the upper, active soil layer, the temperature ranged from about 20 to –35 °C in 2007 [52]. The island is divided into two main geomorphological parts: the low-lying modern floodplain in the western part of the island and the river terrace with a vertical height of 16 m in the eastern part (Figure 1). Both geomorphological units were formed during the Holocene, but the soil material of the modern floodplain is considerably younger and easily degradable because the organic carbon originates from the eroded river material. The river terrace is mainly characterized by low-centered ice-wedge polygons, and the carbon source was built in mosses and is slowly biodegrading because of high CN ratios. The landscape and sites, including the soils of the different geomorphologic units, were described by Sanders et al. [53]. The soils are mainly characterized as Typic Psammorthel and Psammentic Aquorthel according to the international soil classification, in the modern floodplain, and the drier parts of the river terrace, respectively. At the polygons of the polygonal tundra, Typic Aquiturbel was found in the rim and Typic Histothel in the center. In the transition between rim and center—the polygon slope—intermediates of these soil types were found by the soil survey staff in 2014 [54].

### 2.2. Sampling and Investigation Strategy 

The main sampling was done in July 2008. Soil sampling was carried out in different landscape units of Samoylov Island for potential ammonia oxidation measurements, temperature adaptation of nitrification, and investigation of the microbial—especially the nitrifying—community. The active layer had a thawing depth up to approximately 70 cm, and different soil types were sampled in this active layer in two or three different depths (0–5, 5–15, 15–25 cm). We collected three replicates and three sampling points at all sites. At the drier river terrace at the attendant cliff of the Lena River Delta (IS2), two additional permafrost soil samples were taken at depths of 150 and 250 cm below surface level. The main goal of this sample effort was to investigate nitrification in thawing permafrost, which was recently exposed by erosion of the cliff. Further methods and results of the soil characteristics are described elsewhere [53]. To sample the Samoylov cliff (IS3) up to 5 m below the ground surface, the thawed material was mainly removed, and permanently frozen samples were collected [55]. These samples from the Samoylov cliff taken in 2005 and were further used for enrichment cultures.

### 2.3. Ammonia Oxidizing Potentials

The samples taken in 2008 (Table 1) were directly measured for potential nitrification rates using a modified ISO/DIN 15685:2001 standard test [56]. Briefly, 12.5 g of fresh soil from each sample was weighed into 50 mL of a 0.75 mM ammonium sulfate (Merck, Darmstadt, Germany) solution in triplicate. For inhibition of nitrite oxidation, potassium chlorate (Merck, Darmstadt, Germany) was added to an end concentration of 5 mM. To differentiate between bacterial and archaeal ammonia-oxidizing activity, the antibiotic streptomycin (50 µg mL^–1^) (Merck, Darmstadt, Germany) was added in one replicate to inhibit bacterial activity. Bottles were shaken at 125 rpm at in-situ temperature, meaning that the shaker was positioned in a hole in the active layer of the soil outside in the dark. During the time of incubation, the soil temperature was between 0 and 10.5 °C, with an average of 5.2 °C [53]. Potential nitrification activities were measured by nitrite formation over a period of six-weeks in the field. Samples for nitrite measurements in the field laboratories were taken twice a week. The potential ammonia oxidation rates were calculated at the linear and steepest increase of nitrite over time. In the first hours and up to one day, no activity was detectable.

### 2.4. Temperature Adaptation of Soil Incubations

To determine the temperature adaptation of potential ammonia-oxidizing activity, we used a temperature gradient between approx. 0 and 40 °C. In the particular incubations, the temperature gradient slightly differed in this range. This temperature gradient was created in a metal block with 42 boreholes for flasks, using a heater (iTRon 32 (JUMBO®) Fulda, Germany) and a cooler (Julabo F 25, Seelbach, Germany). For the soil activities, we used 2.5 g of dewed soils and 25 mL of phosphate buffer. Replicates or triplicates were inoculated, and the activities were calculated by nitrite accumulation per day. 

### 2.5. DNA Extraction and amoA Analysis of Archaea and Bacteria

The entire DNA of 12 selected dewed soil samples—after frozen transport to Germany—was extracted by using the MoBio PowerSoilKit (MoBio, Carlsbad, CA, USA) applied according to the manufacturer's instructions (see Table 2). The DNA of enrichment cultures was extracted by DNA extraction kit (MoBio, Carlsbad, CA, USA) and subsequently stored at –20 °C.

For this DNA from dewed soils and active enrichment cultures, PCR of the bacterial *amo*A [57] and archaeal *amo*A gene [58] was performed.

### 2.6. Analysis of 16S rRNA Amplicon Sequencing Diversity 

Diversity studies were performed via 16S rRNA amplicon sequencing using the Illumina MiSeq platform of the molecular service-company MR DNA (Shallowater, TX, USA). For this, isolated DNA from 6 dewed soil samples were investigated (see Table 1 and Table 2). The primer sets 519f and 802r were used for preparative PCR, followed by Illumina MiSeq with a sequencing depth of 20,000 reads per sample, at the service facility. Operational taxonomic units (OTUs) were obtained from the preprocessed sequences by the QIIME (v.9.1) [60] de novo OUT picking workflow, summarized and visualized by the QIIME *summarize_taxa_through_plots.py* script. Illumina sequences were processed, classified, and summarized by MR DNA and their analysis pipeline (MR DNA, Shallowater, TX, USA) [61]. In short, sequences were joined and depleted of barcodes, followed by removal of sequences <150 bp or with ambiguous base calls. Sequences were denoised, OTUs were generated, and chimeras were removed. OTUs were defined by clustering at a 3% divergence (97% similarity).

Final OTUs were taxonomically classified using BLASTn against a curated database derived from GreenGenes, RDPII, and NCBI [62]. Relative abundances of microbial taxa were determined as a percent of the total reads for each sample. To assess phylogenetic relatedness between the microbial communities, OTU results were used for a nonmetric multidimensional scaling (NMDS) approach based on Bray–Curtis similarities by using the program package PAST version 2.17 [63]. 

### 2.7. Characterization of Enrichments of Nitrifying Microorganisms

In this study, we investigated enrichment cultures from different soil types from Samoylov Island, which were enriched from soil samples taken in 2004, 2005, and 2008. The enrichment cultures were incubated at different temperatures described in Table 3. In brief, the conditions were as follows: after frozen transport to Germany, ammonia oxidizers were enriched by sieving (2 mm) 1 g dewed soil material and suspending it in 10 mL medium of mineral salts with ammonium chloride (0.5 or 1 mM) (Merck, Darmstadt, Germany) [64]. Incubations were performed at different temperatures (4, 10, 18, and 28 °C) in the dark without shaking. Ammonia consumption was tested by using test strips (Merck, Darmstadt, Germany), and nitrite formation was checked by the Griess and Ilosavay spot test [65]. The upscaling procedure was performed as described by Spieck et al. [66] to obtain a sufficient cell amount for further analysis. Purity tests were carried out modifying the tests of Steinmüller and Bock [67]. During this study, the ammonia-oxidizing microorganisms were highly enriched, but pure cultures were not achieved. 

### 2.8. Molecular Biologic Approaches

For the characterization of enriched nitrifiers, PCRs of the bacterial [57] and archaeal amoA genes [58] were performed, and the microbial communities of the cultures were investigated by DGGE (denaturing gradient gel electrophoresis) approaches [70]. For the analysis of the 16S rRNA, gene primer sets for bacteria [71] and archaea [72] were used, and, afterward, these 16S rRNA gene sequences were compared with those on publicly accessible databases using the Basic Local Alignment Search Tool (BLAST, NCBI) [73]. After the DGGE, the sliced bands were reamplified and sequenced. For sequencing the entire 16S rRNA, the PCR product was directly ligated into the pGEM-T vector cloning system (Promega, Mannheim, Germany) and transformed into competent cells, as described in the manufacturer’s instructions. For partial and near-complete sequencing of clone inserts, the plasmid primers SP6 and T7 were used to reamplify the insert. For the calculation of the phylogenetic tree, we used the program MEGA 7 and for the necessary alignment the program ClustalW [74]. 

### 2.9. Electron Microscopy

In addition to the DGGE approaches, visual identification of the cultivated nitrifiers was also done by electron microscopy. For analyzing the ultrastructure by electron microscopy, cells were collected at 13,000 rpm, fixed with 2.5% (*v/v*) glutaraldehyde and 2% (*w/v*) osmium tetroxide, and embedded in Epon 812 (Serva, Heidelberg, Germany), according to the previously published protocol [66]. Ultrathin sections were examined with a transmission electron microscope (Zeiss model Leo 906E with a CCD camera model 794, Oberkochen, Germany). 

### 2.10. Temperature Adaptation of Enrichment Cultures

To investigate the temperature adaptation of the enrichment cultures, the same testing approach was used as for the temperature adaptation of the soil incubations (see above). For the enrichment, culture flasks with 25 mL of mineral medium [64] were used, which contained ammonium chloride (Merck, Darmstadt, Germany) as substrate (1 mM) and calcium carbonate (Merck, Darmstadt, Germany) as buffer. Ten percent of a constantly growing pre-culture served as the inoculum.

## 3. Results

### 3.1. Potential Ammonia Oxidizing Activities

Potential ammonia oxidation activities were detectable in nearly all investigated soils types of Samoylov Island, varying between geomorphological units, vegetation cover, and soil types (Table 1). In all samples of the sandy soils (Psammorthel and Psammentic Aquorthel according to soil survey staff [54]) of the modern floodplain and the dry river terrace, potential ammonia-oxidizing activities were measured between 100 and 600 ng N g^–1^ dw h^–1^ in the absence of antibiotics. The highest activities were found in the upper layer of the non-vegetated and sand-dominated beach (B0–5) of the modern floodplain and in the recently thawing permafrost samples 2.5 m below the ground surface on the cliff under the dry river terrace (C250). Both samples were characterized by the highest amount of organic matter with 3.9% ± 1.0% and 4.9% ± 2.6% of the non-polygonal tundra soils (Table 2) and missed live plant root, respectively.

In contrast to the sand dominated soils, the ammonia-oxidizing potential was clearly lower in the organic matter-dominated and water-saturated soils of the polygonal tundra (IS1). Ammonia oxidation potential in soils of the polygonal tundra was only detected in the upper layer of the polygonal center (PC0–5) and the subsurface layer (PR5–15) of the polygon rim varying between 30 and 100 ng N g^–1^ dw h^–^1 (Table 1).

When bacteria were inhibited by the antibiotic streptomycin, significant ammonia-oxidizing activity was only detected in the upper layer of the beach with 197 ng N g^–1^ dw h^–1^ (B0–5, Table 2). 

### 3.2. Temperature Adaptation of Potential Ammonia Oxidation in Soils 

**Figure 2:**
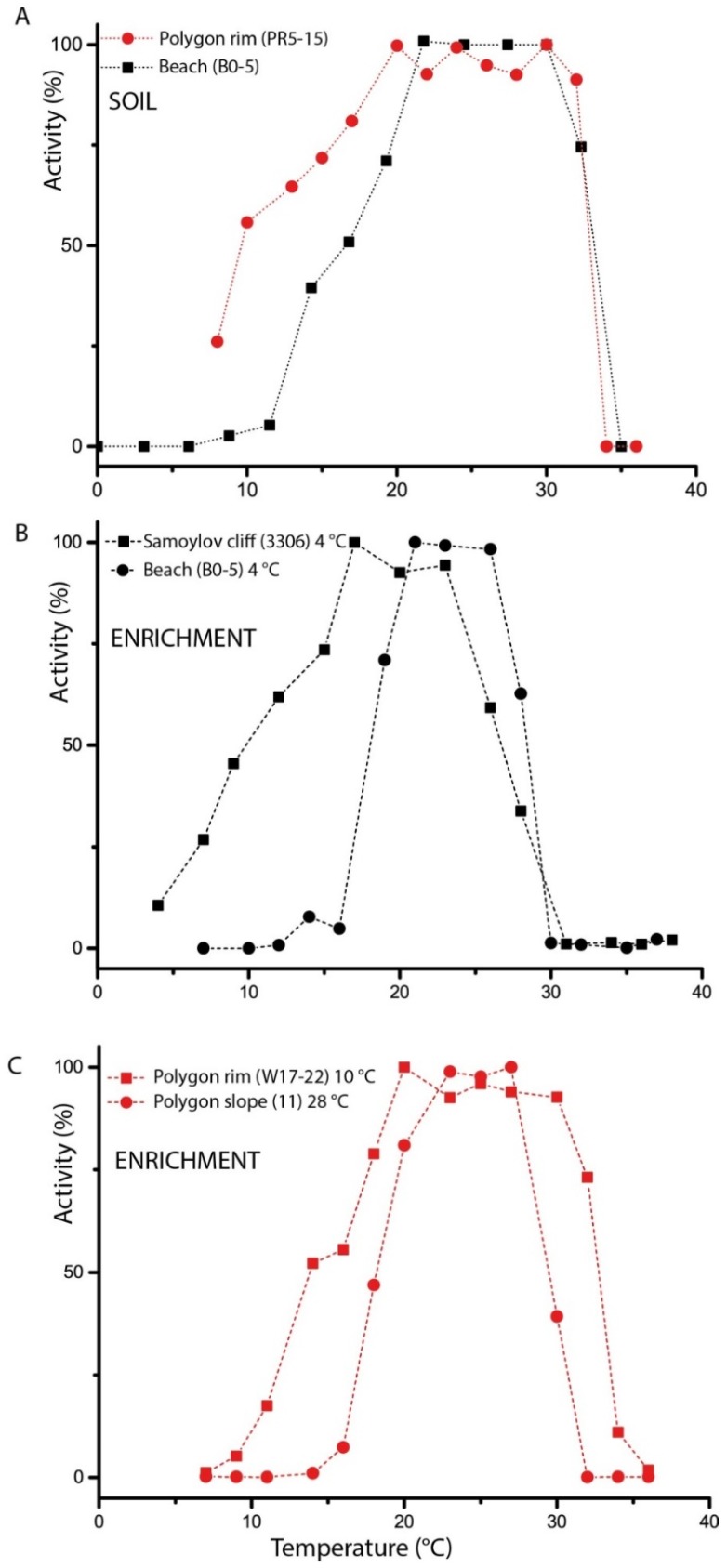
Temperature adaptation of ammonia oxidation in soil samples and enrichment cultures of permafrost-affected soils of Samoylov Island. Black symbol: sand dominated samples, red symbols: samples of polygonal tundra. The highest activity was equalized 100%. (**A**) Soil samples of polygon rim (PR5–15) and beach (B0–5). (**B**,**C**) Highly enriched ammonia-oxidizing cultures (see Table 3).

To determine the temperature spectrum of ammonia oxidation in soil samples of the Arctic tundra, potential ammonia-oxidizing activity rates were measured in temperature gradients between 0 and 36 °C. Soil samples of the organic-rich polygon rim soil (PR5–15) in a gradient from 8 to 36 °C and the sand dominated and non-vegetated beach soil (B0–5) in a gradient from 0 to 35 °C were tested (Figure 2A). The incubations were carried out after the soil samples were transported frozen to Germany.

Potential ammonia-oxidizing activities of the soil samples of the polygon rim 5–15 cm were detected over a range of 8 to 32 °C with a broad optimum between 20 and 32 °C. At 10 °C, the activity still reached above 50% of the optimum activity. The temperature spectrum of ammonia-oxidizing potentials of the beach (0 to 5 cm) was slightly different, with an optimum between 22 and 30 °C. Fifty percent activity was measured at 16 °C, and, below 8 °C, no more activity was detectable. 

### 3.3. Microbial and Nitrifying Community Composition

**Figure 3:**
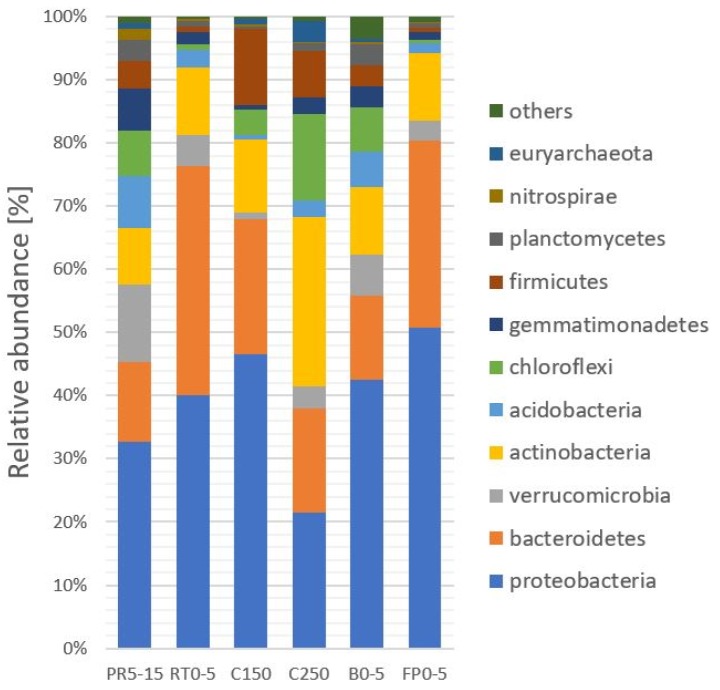
Relative abundance of microbial phyla in soils of Samoylov Island. Polygonal rim (PR5–15); dry river terrace (RT0–5); cliff (C150), cliff (C250); beach (B0–5); floodplain (FP0–5) (Table 3).

The 16S rRNA based microbial community analysis of the soil samples revealed a high diversity at the phylum level with Proteobacteria, Bacteriodetes, Actinobacteria, and Chloroflexi as dominant groups (60% and 90% relative abundance, Figure 3). OTUs classified as nitrifying taxa revealed a relative abundance of 6.2% of the total reads in the sample from the polygon rim (PR5–15) and 0.6% in the sample of the floodplain (FP0–5) (Table 2).

**Figure 4:**
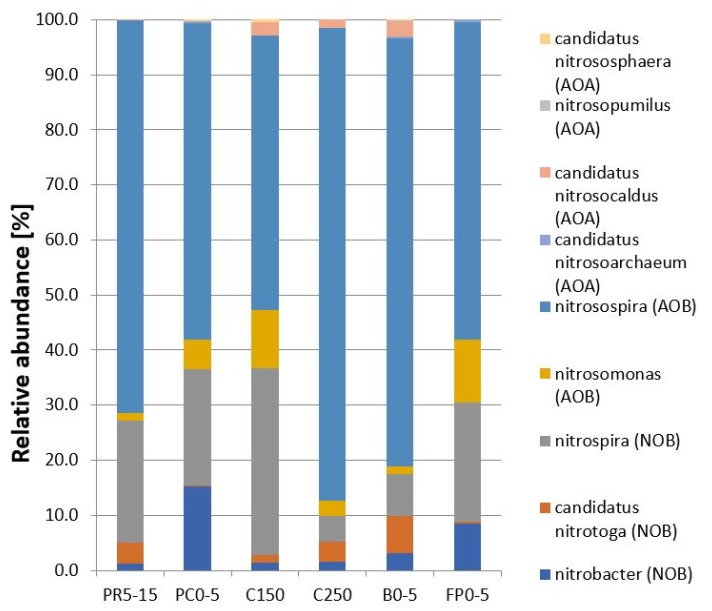
Relative abundance of known nitrifying microorganisms on the genus level in soils of Samoylov Island. Polygonal rim (PR5–15); dry river terrace (RT0–5); cliff (C150), cliff (C250); beach (B0–5); floodplain (FP0–5) (Table 3).

*Nitrosospira* as AOB could be identified as the main nitrifier with a relative abundance of 50% to 80% of the reads classified as nitrifiers (Figure 4). *Nitrospira* were the predominant NOB with up to 34% as nitrifier reads, e.g., in the cliff (C150). Only a few AOA could be detected with a relative abundance between 0.2% and 3.4% of all nitrifier reads (Figure 4).

**Figure 5:**
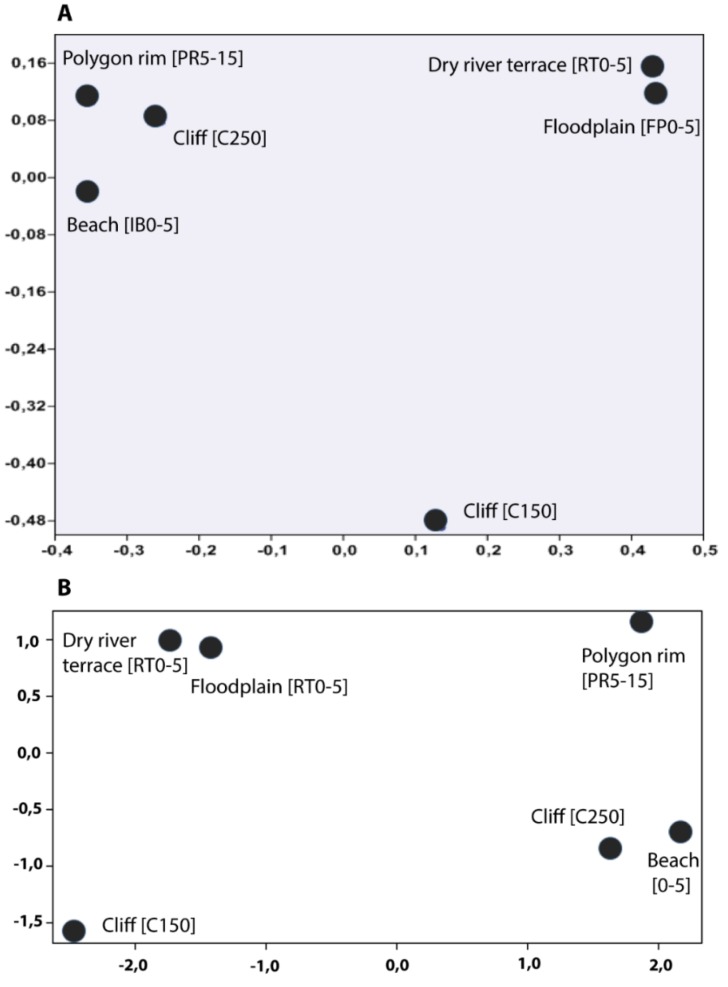
Cluster analysis of microbial community (**A**) and soil parameter (**B**). A: non-metric multidimensional scale (NMDS) based on Bray–Curtis similarities of the nitrifier OTUs. B: metric multidimensional scale (MDS) of the soil parameters presented in Sanders et al. [53].

To assess the phylogenetic relatedness between the microbial communities from the different sampling sites, a non-metric multidimensional scaling (NMDS) based on Bray–Curtis similarities was applied (Figure 5A). To see a clustering of the soil types, a metric multidimensional scaling (MDS) of soil parameters was done. For parameters such as organic matter content, CN ratio, pH, DIN concentration, water content, and potential ammonia-oxidizing activities were used (Figure 5B, Table 1). The resulting 2D ordination plots revealed that the samples clustered in two groups. The organic-rich, aerobic samples from the polygon rim (IS1), beach (IS 4), and the cliff 250 (IS2) versus a second group, which was characterized by dry sandy soils with low organic matter at the floodplain (IS5) and dry river terrace (IS2). The cliff 150 (IS2) could not be assigned to any of the two groups.

### 3.4. Detection of amoA-genes in Soil Samples 

To determine the occurrence of ammonia-oxidizing bacteria and archaea, PCR targeting the gene of ammonia monooxygenase (*amoA*) subunit A was performed (Table 2). The bacterial *amoA*-gene was detected in all soil samples except the organic-rich and water-saturated soil of the polygon center. In contrast, the *amoA*-gene of archaea was only detected in soils of the geomorphological unit modern floodplain including soils of the non-vegetated beach and the vegetated soil of the floodplain. At the geomorphological unit river terrace, archaeal *amoA* was only found at the drier part of the river terrace. 

### 3.5. AOA in Permafrost-Affected Soils

**Figure 6:**
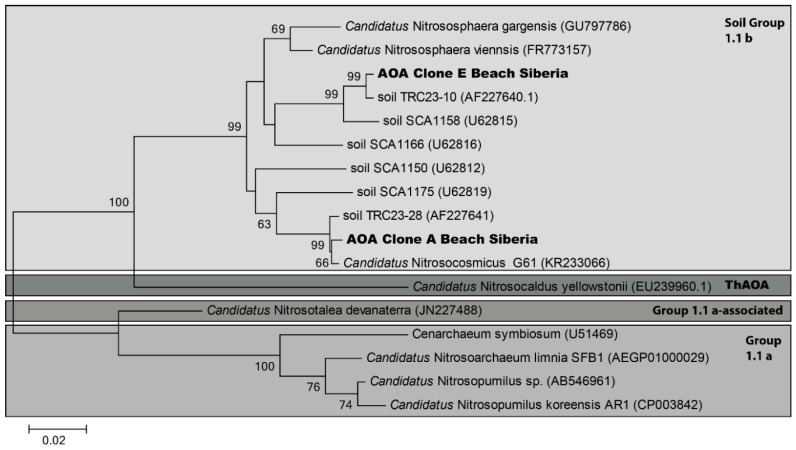
Maximum-likelihood tree depicting the phylogenetic relationships between the archaeal 16S rRNA gene sequences of cloning products of soils from the beach and other *Thaumarchaeota*-like sequences in group 1a + b and selected cultured representatives. The tree was constructed using sequences of ±900 b.p. Nodes supported by bootstrap values are indicated. Scale bar = 2% sequence divergence.

The archaeal *amoA*-gene was found by PCR in the upper soil sample of the beach, and ammonia-oxidizing activity was detectable in the presence of streptomycin (Table 1). Therefore, we analyzed the archaeal community of the samples from the beach (IS4) more intensively. For further investigation of AOA, 16S rRNA sequencing was performed. As shown in the resulting phylogenic tree (Figure 6), the two AOA clones (A and E) were grouped into the 1.1b soil group. Both clones had a sequence similarity of the 16S rRNA gene of 94% to *Candidatus* Nitrososphaera gargensis, which was originally isolated from the Baikal-region [75], as well as *Nitrososphaera viennsis*, which was isolated from soil in Austria [76]. Clone A showed a similarity of 99% to the recently published *Candidatus* Nitrosocosmicus originating from a municipal wastewater treatment system [39].

### 3.6. Identification of Ammonia-Oxidizing Microorganisms in Enrichment Cultures 

**Figure 7:**
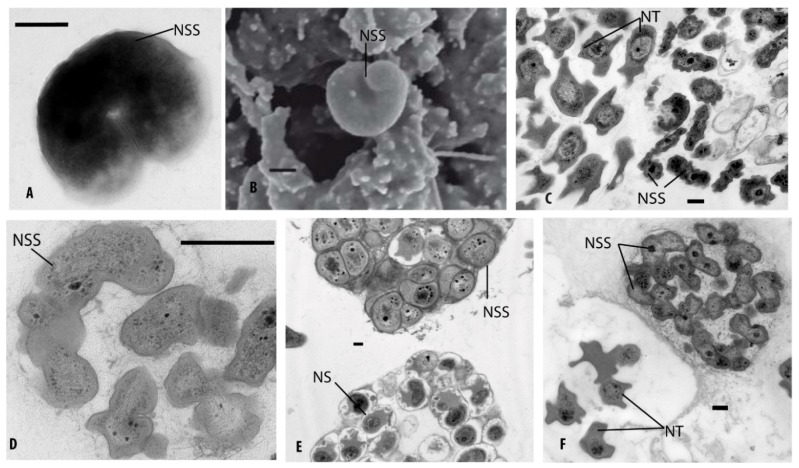
Electron microscope pictures of enrichment cultures of ammonia and nitrite-oxidizing bacteria (AOB and NOB) from soils from Samoylov Island. (**A**,**B**) Visualization of total cells. (**C**–**F**) Electron micrographs of ultrathin sections. (A–C) Nitrifying enrichment cultures derived from soils of Samoylov cliff (3306 A + B; 3304 C) with *Nitrosospira*-like and *Nitrotoga*-like cells. (D,E) Soils of polygonal tundra, rim (W17–22 cm) and slope (12–17 cm), *Nitrosospira* and *Nitrospira* microcolonies. (F) Beach soils (B0–5) with *Nitrosospira*-like and *Nitrotoga*-like cells. Bars = 0.2 µm, NSS: AOB *Nitrosospira*, NS: NOB *Nitrospira*, NT: NOB *Nitrotoga*, **A**: negatively stained image, **B**: scanning electron microscopic (SEM) image, **C–F**: transmission electron microscopic (TEM) images.

Bacterial *amoA*-genes of AOB were present in all enrichment cultures (Table 3). However, archaeal *amoA* genes could be only detected in the enrichment culture of permafrost samples of the Samoylov cliff (IS3, 3306) and soil samples of the beach (B0–5). In all enrichment cultures, the presence of *Nitrosospira*-like AOB could be confirmed by DGGE, and, in addition, by transmission electron microscopy by their spiral cell form (Figure 7, Table 3). 

In the enrichment cultures, nitrite-oxidizing bacteria could be successfully grown in coexistence with ammonia-oxidizing microorganisms (AOM). Based on their particular morphology, they could be identified as *Nitrotoga*-like as well as *Nitrospira*-like organisms (Figure 7C,E,F).

**Figure 8:**
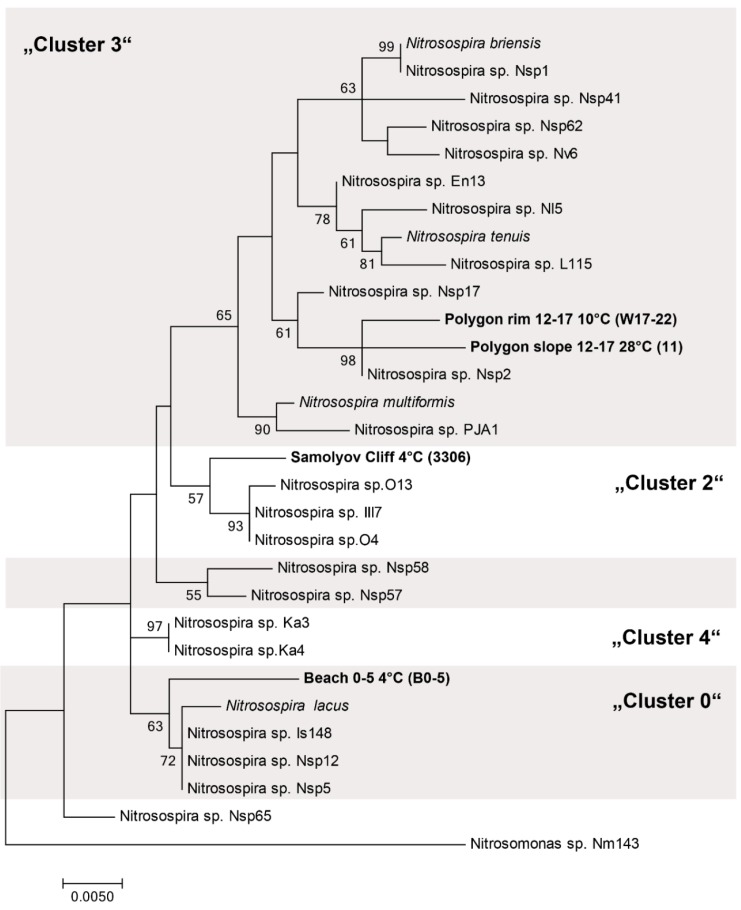
Maximum-likelihood tree based on phylogenic relationships between the bacterial 16S rRNA gene of known *Nitrosospira* and *Nitrosospira*-like bacteria enriched in this study. The tree was constructed using sequences of ±1400 b.p. Nodes supported by bootstrap values are indicated. Scale bar = 5% sequence divergence. Tree is based on Purkhold et al. [77].

The cloned 16S rRNA gene sequences of the enrichment cultures were used to create a maximum-likelihood tree of AOB based on the 16S rRNA sequence analysis of Purkhold et al. [77] (Figure 8). All four clones were affiliated with *Nitrosospira*; both sequences of the polygonal tundra belonged to Cluster 3 and the clone from Samoylov cliff to Cluster 2. The *Nitrosospira-*like 16S rRNA of the enrichment from the cliff was grouped in *Nitrosospira* Cluster 2 and had a high similarity to sequences of *Nitrosospira* found in other cold environments, e.g., soils in Norway (Figure 8). The *Nitrosospira*-like enrichment from the organic-rich layer of the beach belonged to Cluster 0, which included the psychrotolerant *Nitrosospira lacus* isolated from a sandy lake sediment [35]. 

Otherwise, both *Nitrosospira-*like bacteria enriched from the polygon tundra belong to *Nitrosospira* Cluster 3, which contains species with high natural abundance. Although the cultures were enriched at different temperature (10 and 28 °C), these *Nitrosospira* cultures were closely related. In contrast, the *Nitrosospira*-like bacteria enriched from the beach belonged to the Cluster 0, which contains mainly bacteria from soils.

### 3.7. Temperature Adaptation of Ammonia Oxidation in Enrichment Cultures

To determine the temperature dependence of ammonia oxidation, the enrichment cultures from permafrost samples of the Samoylov cliff (3304/3306) and non-vegetated beach soil (B0–5) were tested (Figure 2B); both were grown at 4 °C. Additionally, two enrichment cultures from the polygonal tundra (IS1 incubated at a higher temperature (10 and 28 °C) were used (Figure 2C). 

The enrichments grown at 4 °C showed different temperature sensitivities (Figure 2B). The temperature optimum of the enrichment culture from permafrost samples from the Samoylov cliff was 17 °C, and, above 20 °C, the cells remained active. At 10 °C, the activity of the cliff sample was still above 50%. 

In contrast, the enrichment culture from the polygonal tundra, the rim and slope preincubated at a higher temperature of 10 and 28 °C, respectively, were less adapted to cold temperatures (Figure 2C). Both had the optima at approximately 20 °C, but the temperature spectra of the enrichment from the polygon slope was narrower, and no activity was found above 30 °C. The enrichment from the polygon rim had a very wide spectrum and a maximum clearly above 30 °C.

The enrichment cultures, which contained *Nitrosospira*-like bacteria (Samoylov cliff and polygonal tundra), could be described as cold-adapted, but according to the definition of Morita [78], they were not psychrophilic. The enrichment culture from the beach, however, enriched at 4 °C, showed a mesophilic temperature spectrum.

## 4. Discussion

The investigation of the various soil types of cryosols of Samoylov Island regarding the microbial diversity and nitrification, including canonical nitrifiers, demonstrated that the nitrifying community is an important component of the microbial community in the ecosystem and varies over small scales depending on soil properties. Phylogenetic analysis based on the DNA level, and enrichment approaches identified the AOB *Nitrosospira* as potential crucial ammonia-oxidizing microorganisms in this ecosystem, although direct evidence for their activity is still missing. Despite the low N-availability and extreme environmental conditions, nitrification continued and varied depending on substrate availability and oxygen supply, which was in turn mediated by the quality of the organic matter and water content. In consequence, the temperature is the most important factor to control nitrification. The nitrification rates of soils from the polygonal tundra and the beach were adapted to the cold environment as well as the enrichment culture were adapted, but were not cryophilic. Further on, *Nitrosospira* were best adapted to extreme environmental conditions, including slightly acidic pH in the soils. 

### 4.1. Potential Ammonia Oxidizing Activities in Permafrost-Affected Soils

The main driving factor for nitrification in the investigated cryosoils was the temperature regime and the very short vegetation period, due to the existing extreme temperature gradient in these soils during the year. The measured potential rates in the soils of Samoylov Island were between 0.1 and 0.5 µg N g^–1^ dw h^–1^. Alves et al. [25] measured higher potential rates in high Arctic soils of Svalbard, from 2 up to 150 µg N g^–1^ dw h^–1^, by using the isotope dilution method and a temperature of 15 °C. However, Hayashi et al. [24] found significantly lower values in the same area with 1.1 to 14.1 ng N g^–1^ dw h^–1^. To understand the wide range, the methodological differences, including incubation temperature, have to be kept in mind. Hayashi at al. [24] used the same method as in the present study at a temperature of 10 °C, but nitrite/nitrate increase was only measured in the first 8 to 24 hours. During this time period, we did not see an increase in our incubations, and in consequence, our experiments were extended to 6 weeks in total. In addition, Alves et al. [25] did not detect net nitrification rates during their short-term incubations. For the incubations, they used the isotope dilution method and reported high gross rates. 

However, rates of a similar magnitude were found in Alaska, Greenland, and other parts of Siberia. For example, Petersen et al. [21] found potential nitrification rates across a vegetation gradient in Alaska with 75 and 470 µg N g^–1^ h^–1^. Wild et al. [79] found gross nitrification by isotope dilution of 20 to 170 µg N g^–1^ h^–1^ in Greenland and Siberia. Both investigations sites were much more south than Samoylov Island, with higher average temperatures. Nevertheless, it has to be considered that potential nitrification did not represent the in-situ activity, and the measurement conditions were often different. Further, the soil types varied strongly in the investigated areas. Indeed, our net rates showed that ammonium was potentially oxidized during the vegetation period; however, significant amounts of ammonium and low amounts of nitrate were found in these soils [53]. Thus, the substrate ammonium was evidently not a limiting factor for nitrification. 

### 4.2. Occurrence of AOB and AOA in Permafrost-Affected Soils

According to the potential ammonia-oxidizing activities, ammonia-oxidizing microorganisms (archaea and bacteria: AOM) were detected in most of our study sites in permafrost-affected soils of Samoylov Island. Reports about the distribution of AOB and AOA in cold regions are inconsistent. In soils of the Antarctic, the bacterial *amoA* gene predominated over the archaeal *amoA* gene, as reported by Jung et al. [80]. Boyd et al. [81] detected archaeal amoA genes in a subglacial ecosystem in Canada. In contrast to the present study, they showed a lower abundance and they were not as diverse as those of AOB in soils of Samoylov Island. Similar to the activity, the occurrence of AOA vs. AOB in permafrost soils of Svalbard was inconsistent. Alves et al. [25] found a widespread distribution of diverse AOA throughout a variety of distinct tundra soils. In contrast to that, AOB were often undetectable. Hayashi et al. [24] found both AOB and AOA in the soils of Svalbard. However, it has to be kept in mind that different primer-sets were used and that our PCR for AOA was conducted with primers from Francis et al. [58]. More recently, primer sets with a broader spectrum have been developed [25,76]. 

The remaining question is, which permafrost niches were occupied by AOA and AOB? *Nitrosospira*, as an AOB, as well as AOA, are adapted to a low availability of substrate and cold environments [31]. Consequently, the particular small spatial differences and niches must be specified. The pH could be the crucial parameter for the differentiation. Aigle et al. [48] addressed the question if the pH was a key factor of ecological distribution. Some AOA are adapted to lower pH than AOB [34,82]. In cold environments, AOB seem to be better adapted to slightly acid soils such as those reported by Boyd et al. [81] and Hayashi et al. [24]. 

### 4.3. Microbial Communities 

As shown in previous studies, bacteria are more abundant and diverse in permafrost-affected soils compared to archaea or fungi [83]. This feature is confirmed in our study of Samoylov Island with Proteobacteria, Bacteriodetes, Actinobacteria, and Chloroflexi being the most abundant groups. Liebner et al. [84] detected mainly Bacteriodetes and Actinobacteria in soils of the polygonal tundra, while Frey at al. [85] found a similar microbial community in alpine permafrost soils in accordance with our data. Similar distributions were presented in Malard and Pearce [19], including changes in the structure of Bacteriodes and Actinobacteria between different regions of the Arctic. 

On a genus level, *Nitrosospira* is the main representative of ammonia oxidizers in the soils of Samoylov Island. The highest proportions, with 4.8% and 6.3%, respectively, were found in soils of the beach and the polygon rim, where high activities of nitrification were also detected. Both samples clustered together with the sample from the cliff (250 cm) due to the similar organic matter content and CN ratio (Figure 5). 

### 4.4. Nitrosospira: the Primary Enriched AOB Representative

In this study, *Nitrosospira* was the main genus of ammonia-oxidizing bacteria enriched from permafrost-affected soils of Samoylov Island, whereas a high enrichment of AOA with our cultivation methods was not obtained. *Nitrosospira*-like bacteria, detected in all analyzed soils and enrichment cultures, were characterized by molecular as well as microscopic methods. Their morphological appearance varied from short curved rods to long coiled spirals, and some cells were surrounded by extracellular polymeric substances (EPS), as is typical for most nitrifiers (Figure 7). Such morphological variability was already described for *Nitrosospira,* depending on the surrounding conditions and their fitness [28,35,86].

Several environmental factors are potentially responsible for the predominance of *Nitrosospira* in the soils of Samoylov Island. For example, *Nitrosospira* species have been enriched and isolated from soils with low pH values [28,87]. Although the pH values of the investigated soils in Siberia were neutral to only slightly acidic, they might have been selective to enhance the growth of *Nitrosospira*. Additionally, Avrahami et al. [88] found a correlation between low temperature and the occurrence of *Nitrosospira*. In a cold mining operation retention pond in the Northwest Territories, Canada, *Nitrosospira* was identified as the main AOB [89]. In addition, Hayashi et al. [24] detected AOB belonging to the genus *Nitrosospira* in Svalbard; however, cold bioreactors treated with inorganic mine waters contained *Nitrosospira* together with *Nitrosomonas* [90]. The sequencing approach was also used to identify several OTUs assigned to *Nitrosomonas*, especially in the floodplain and at the cliff, with up to 10% of the nitrifiers (Figure 4). Here, the influence of the substrate concentration seemed to be ambiguous. At our investigation site, the combination of slightly acidic pH and low in situ soil temperatures with a maximum of 10 °C and the low available substrate might have supported the dominance of *Nitrosospira*-like ammonia-oxidizing bacteria.

### 4.5. Which Nitrifiers are Adapted to the Extreme Environment of Low Temperature?

So far, ammonia-oxidizing bacteria have been described as being mesophilic [29], except *Nitrosomonas cryotolerans*, which is able to grow at temperatures down to –5 °C [91], and *Nitrosospira lacus*, which can grow at 4 °C [35]. However, the temperature optimum of *N. cryotolerans* is either 22 °C or up to 30 °C, depending on the incubation conditions of the precultures (5 or 25 °C) [92]. In general, ammonia oxidizers of marine habitats are better adapted to cold conditions compared to those in terrestrial habitats. In marine sediments in Svalbard, Norway, nitrification exhibited a temperature optimum of 14 °C [93], indicating that this process was accomplished by psychrophilic microorganisms. Unfortunately, the examined active ammonia oxidizers from Svalbard were not cultivated and their physiological features still remain unknown. In coastal sediments, an unknown AOB enrichment derived from in situ temperatures of 3 to 12 °C revealed a moderate temperature optimum for nitrification above 22 °C [94]. As shown by molecular methods, in cold arctic marine habitats, mainly AOB of the genus *Nitrosospira* were detectable [95]. This indicates that representatives of this phylogenetically young genus are well adapted to the conditions of cold environments. In contrast, no cold-adapted AOA have been described so far, although they were enriched from the soils of Svalbard [25] and also detected in the ocean at low temperatures [96]. In soils in temperate climates, AOA have shown a 12 °C higher temperature optimum over AOB [97]. 

At our investigation site, soils were frozen for most of the year, and during the vegetation period, the active layer did not exceed temperature over 10 °C and had an average value of 4.1 °C [53,98]. Our results showed that the enriched ammonia-oxidizing bacteria were not psychrophilic but could be characterized as psychrotolerant. However, existing definitions of the temperature adaptation are difficult to correlate with physiological features. By definition, psychrophilic organisms have a temperature optimum below 15 °C and a maximum below 20 °C [78]. Microorganisms showing a mesophilic temperature spectrum but still having a high activity below 4 °C were termed as psychrotolerant. Interestingly, the temperature spectrum of the AOM in soils as well as in the corresponding enrichment cultures of the polygons and the floodplain beach showed different ranges. The water regime of the Lena River seems to have a strong influence on the temperature regime and the composition of the microbial community. 

The accompanying NOB *Nitrotoga arctica* [99], obtained from the same habitat, was characterized as a moderate psychrophilic bacterium with a temperature optimum of 13 °C (unpublished result), whereas permafrost-members of *Nitrospira* are considered mesophilic NOB [100]. It remains unclear why cold adaptation of AOB in permafrost-affected soils is unincisive, since their temperature optimum is clearly above 20 °C. Although *Nitrosospira* is enriched at 4 °C, the existence of further, although so far unseen, psychrophilic AOB cannot be excluded. Generally, most microorganisms existing in Arctic soils are not extremophilic but psychrotrophic [19].

## 5. Conclusions

This study shows that nitrification plays an important role in permafrost-affected soils of Samoylov Island, although it is generally assumed that low nitrogen availability causes low nitrification rates. Due to the permafrost, the investigated soils can be considered as an extreme environment, and the results suggest active turnover of nitrogen occurs mainly in the very short vegetation period. As a potential crucial ammonia oxidizer in the soils of Samoylov Island, *Nitrosospira* was identified by molecular and cultivation-based analysis accompanied by the nitrite oxidizer *Nitrospira*. 

The measurements of in situ nitrification rates, quantification of active AOB, AOA, and comammox, and the physiology of the involved microorganisms can help to increase our knowledge of the permafrost ecosystem. The water of the Lena River strongly influences nitrogen turnover in the floodplain, as the river connects the warmer southern region to the cold arctic environment. Further research is needed on the impact of climate change on the adaptation of the nitrifying community and their role in N_2_O production and emission.

## Figures and Tables

**Table 1:** Site information for the sampling in July 2008.

Sampling Site	Sample name	Depth (cm)	Organic matter (%)	pH (H_2_O)	Corg (%)	N (%)	CN	NH_4_^+^ (g g^–1^ dw)	N0_2_^-^ (g g^–1^ dw)	NO_3_^-^ (g g^–1^ dw)
**“River terrace”**										
Polygon rim (IS1)	PR0–5	0–5	17.1	6.6	10.0	0.3	37	0.3	0.02	bdl
Polygon rim (IS1)	PR5–15	5–15	5.3	6.6	3.1	0.2	18	0.3	bdl	bdl
Polygon rim (IS1)	PR15–25	15–25	4.9	6.2	2.9	0.2	16	0.1	bdl	bdl
Polygon center (IS1)	PC0–5	0–5	37.1	6.3	18.6	0.6	33	0.5	0.01	bdl
Polygon center (IS1)	PC5–15	5–15	36.1	6.1	18.0	0.5	37	0.2	0.01	bdl
Polygon center (IS1)	PC15–25	15–25	13.0	6.0	6.5	0.2	37	0.6	bdl	bdl
Dry river terrace (IS2)	RT0–5	0–5	1.3	6.4	0.8	0.1	17	0.3	0.01	bdl
Dry river terrace (IS2)	RT5–15	5–15	1.1	6.4	0.7	0.1	14	0.0	0.01	bdl
Cliff^*^ (IS2)	C150	150	1.0	6.8	0.6	0.0	15	5.5	0.03	0.1
Cliff^*^ (IS2)	C250	250	4.9	6.9	2.9	0.2	16	10.0	0.02	bdl
**“Modern floodplain”**										
Beach* (IS4)	B0–5	0–5	3.9	6.9	2.3	0.2	15	9.4	0.04	bdl
Beach* (IS4)	B10–20	10–20	0.3	7.0	0.2	0.0	12	0.3	0.02	0.2
Floodplain (IS5)	FP0–5	0–5	1.6	7.3	0.9	0.1	14	0.1	bdl	0.1
Floodplain (IS5)	FP5–15	5–15	1.8	7.3	1.0	0.1	16	0.2	bdl	0.1

bdl: below detection limit, * without vegetation.

**Table 2:** Ammonia-oxidizing potentials and PCR detection of the bacterial and archaeal ammonia monooxygenase subunit A gene (*amoA*) in the investigated soil samples.

Sampling Site		Ammonia Oxidizing Capacities (ng N g^–1^ h^–1^)		Detection of *amo*A by PCR		Microbial Diversity	
				Bacterial ^2^	Archaeal ^2^	Reads	Nitrifiers
		Total ^1^	Plus Streptomycin			Total	%
**“River terrace”**							
Polygon rim (IS1)	PR0–5	bdl	bdl	+	–	nd	nd
Polygon rim (IS1)	PR5–15	114 ± 8	bdl	+	–	87,419	6.25
Polygon rim (IS1)	PR15–25	bdl	bdl	+	–	nd	nd
Polygon center (IS1)	PC0–5	33	bdl	–	–	nd	nd
Polygon center (IS1)	PC5–15	bdl	bdl	–	–	nd	nd
Polygon center (IS1)	PC15–25	bdl	bdl	–	–	nd	nd
Dry river terrace (IS2)	RT0–5	103	bdl	+	+	107,960	1.35
Dry river terrace (IS2)	RT5–15	78 ± 8	3	+	+	nd	nd
Cliff^3^(IS2)	C150	197 ± 11	bdl	+	–	79,183	0.81
Cliff^3^(IS2)	C250	571 ± 113	bdl	nd	nd	71,803	1.76
**“Modern Floodplain”**							
Beach^3^ (IS4)	B0–5	542 ± 13	197	+	+	84,338	4.77
Beach^3^ (IS4)	B10–20	102 ± 8	bdl	nd	nd	nd	nd
Floodplain (IS5)	FP0–5	208 ± 3	6	+	+	103,120	0.66
Floodplain (IS5)	FP5–15	225 ± 6	2	+	+	nd	nd

nd: not determined, bdl: below detection limit. (^1^) Standard deviation of replicates. (^2^) Primer set of Francis et al. [58] and Rotthauwe et al. [59]. For these PCRs, DNA of *Candidatus* Nitrososphaera gargensis and *Nitrosospira sp*. were used as positive and negative control, respectively. (^3^) Without vegetation, + detected, – not detected. Cliff was located below the dry river terrace, which is different from the Samoylov cliff in Figure 1.

**Table 3:** Characterization of ammonia-oxidizing enrichment cultures obtained from permafrost-affected soils.

Enrichment Culture	Sampling Site	Depth (cm)	Year Enriched	Incubation Temperature	DGGE Analyses of 16S rRNA or EM images		Detection of *amo*A by PCR	
					AOB	NOB	Bacteria	Archaea
3304/3306	Samoylov Cliff (IS3)	240/350	2004	4 °C	*Nitrosospira*	Nitrotoga*	+	(+)
W0–5	Polygon rim (IS1)	0–5	2004	4 °C	*Nitrosospira*	-	+	–
W12–17		12–17	2004	10 °C	*Nitrosospira*	-	+	–
11	Polygon slope (IS1)	12–17	2005	28 °C	*Nitrosospira*	*Nitrospira*	+	–
B0–5	Beach (IS4)	0–5	2008	4 °C	Nitrosospira*	*Nitrotoga*	+	(+)
CT0840		0–5	2008	18/28 °C	-	-	+	+

AOB: ammonia-oxidizing bacteria; NOB: nitrite-oxidizing bacteria; * only by electron microscopy (EM) images; + detectable; – not detectable; (+) only temporally detectable. Description of initial samples found in Diploma Thesis and Ph.D. Thesis elsewhere [55,68,69].
